# Egg retention and dispersal activity in the parasitoid wasp, Trichogramma principium

**DOI:** 10.1673/2006_06_16.1

**Published:** 2006-09-01

**Authors:** S. Ya Reznik, N. Z. Klyueva

**Affiliations:** 1 Zoological Institute, St.Petersburg, 199034, Russia; 2 Pavlov Institute of Physiology, St.Petersburg, 199034, Russia

**Keywords:** egg parasitoid, behavior, ecology, physiology, oviposition, movement, Sitotroga cerealella

## Abstract

Effects of egg retention on movement and dispersal activity of Trichogramma principium (Hymenoptera, Trichogrammatidae) females were investigated under laboratory conditions. Individual females were observed during one minute in the absence of hosts. Movement activity and dispersal rate were estimated by the length of the track and by the distance from the start point, respectively. Before the test, all wasps during 2 – 4 days were presented with a possibility to parasitize a factitious laboratory host, Sitotroga cerealella Oliv. (Lepidoptera, Gelechiidae). Wasps that had parasitized before the test show significant reduction of spontaneous walking activity and dispersal rate when compared with females that refused to parasitize the non-preferred host (i.e. manifested egg retention). This effect cannot be considered as a direct arrestment reaction to the host because during the test period, no hosts were provided. Thus, egg retention results not only in temporal spread, but also in more intensive spatial dispersal of a group of simultaneously emerged females.

## Introduction

Egg parasitoids of the genus Trichogramma are employed worldwide for the biological control of insect pests (Smith, 1996), and are also widely used as model insects for research. It has been demonstrated that although factitious host eggs were readily accepted by certain Trichogramma females, other wasps of the same laboratory line delayed parasitization for a period up to 10–12 days. In relatively short-term experiments, this egg retention is manifested as “refusal to oviposit” (Monje *et al.* 1999; Silva and Stouthamer 1999;Carrière and Boivin 2001; Hoffmann et al. 2001; Bennett *et al.* 2002; Hansen and Jensen 2002, see Reznik *et al.* 1998, 2001 for earlier references). Dissections and direct behavioral observations have showed that these females had a lot of mature ovarial eggs but parasitization was blocked at the stage of arrestment and host recognition (Pavlik 1993; Reznik *et al.* 1997,^1998^, ^2001^).

Delayed oviposition in the presence of non-preferred hosts was also recorded in other parasitoids (Donaldson and Walter 1988; Tepedino 1988; Kim 1999; Beck *et al.* 2001) and phytophagous insects (Withers *et al.* 2000). As far as we know, in none of these studies were the relationships between dispersal and reproduction investigated.

Our earlier observations (Reznik and Umarova 1991) suggested that the percentage of time spent in movement by Trichogramma females delaying oviposition was not lower, or only slightly higher, than that in ovipositing wasps. However, in that study movement activity was recorded with the host present. Thus, the reduction of time spent in movement could be caused by time expenditure for parasitization. Besides, host stimuli obviously had a strong direct influence on the female’s behavior (Gardner and Lenteren 1986; Nordlund 1994; Schmidt 1994), while just spontaneous locomotor activity is usually considered as a measure of dispersal (Dingle and Winchell 1997).

The objective of the present research was to evaluate the influence of egg retention on spontaneous movement and dispersal activity in Trichogramma principium Sugonjaev & Sorokina (Hymenoptera, Trichogrammatidae).

## Materials and Methods

In all experiments, we used a laboratory strain of T. principium, collected in the Chimkent district of Kazakhstan from Noctuidae eggs and cultivated for more than 100 generations on the eggs of the grain moth, Sitotroga cerealella Oliv. (Lepidoptera, Gelechiidae).

The laboratory strain was reared and all experiments were conducted at 20°C under a photoperiod of 18 : 6 L:D and humidity about 75% RH. Emerging T. principium adults were offered a possibility to mate during 24 h in large (100 x 30 mm) test tubes with several hundred individuals (males constituted 30–40%). Previous (unpublished) studies have showed that practically all females actually mated, as demonstrated by the presence of daughters in their progeny. After mating, females were placed individually into small (40 x 5 mm) test tubes. Honey (50% aqueous solution) was streaked on the glass to feed the wasps. Eggs (50 – 60) of the host S. cerealella were presented to each female on a paper strip. All females were presented with a possibility to parasitize for 48 h. Then about a half of females were subjected to the test for dispersal and movement activity. In the rest of females, cards with host eggs were taken out of the test tubes, new portions of fresh grain moth eggs were presented for another 48 h, and then the test for dispersal and movement activity was conducted. Thus, half of females were tested when they were 4 days old (after 2 days of contact with hosts), and other females, were 6 days old (after 4 days of contact with hosts).

To test dispersal and movement activity, each female was placed on a arena consisting of a 5 x 5 mm square of paper. Insects were neither anesthetized nor chilled prior to the test. Wasps were released by gentle shake. Each wasp was released in the middle of two concentric circles (1.5 and 5 cm in radius). In all tests the temperature was 20 ± 1°C, the light was provided by 20 W Day Light lamp placed horizontally 30 cm above the arena. To minimize the influence of circadian behavioral rhythms (e.g. Suverkropp et al. 2001), all tests were conducted during 2 to 5 h after the lights on. To exclude the influence of chemical markers left by females, each female was tested on a new arena. Movement of each female was observed during one minute or until it crossed the large circle. The following parameters were recorded:

The mean number of square borders crossed per one second of observation, which was taken as an estimation of movement (walking) activity.
Exit from the small and large circle, which was taken as an estimation of dispersal activity.

The difference between these two parameters is that the first estimates the length of the track (which may be curved) while the second estimates the increase in straight-line distance walked from the starting point.

After completion of progeny development the number of darkened (successfully parasitized) grain moth eggs was taken as the approximate number of T. principium eggs laid because T. principium oviposition in  S. cerealella eggs usually results in the laying of a single egg per host. All studied females were *post facto* divided in two groups: parasitized and manifested egg retention. When analyzing results, female age and pre-test parasitization were taken as factors, with the behavioral parameters described above, as dependent variables.

Five replicates were conducted with different generations of the laboratory strain.Table 1 shows the sample sizes. Because of the high inter-generational variability of T. principium (Schmuck *et al.*. 1996) generation was also taken as a factor. Preliminary treatment of the results showed that distribution of the number of square borders crossed per one second of observation was close to normal in females that parasitized and females that manifested egg retention. Thus, ANOVA , Tukey test, and Pearson correlation coefficient were used for statistical treatment of this variable. For the categorical variable (exit from circles) the Mantel-Haenszel chi-square test was used. This test is used to determine the association between two binary variables controlling for a third (stratification) variable. In our study, pre-test parasitization and exit from circles were binary variables while replicate (generation) was taken as a stratification variable. All statistical procedures were made with SYSTAT 10. 2.

**Table 1:**
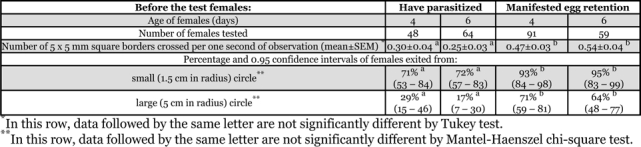
Movement and dispersal activity of Trichogramma principium females in relation to previous parasitization or egg retention.

## Results

Parasitizing females constituted 35% and 52% of the population, and laid 31.6 ± 6.6 (16 – 44) and 39.9 ± 13.1 (14 – 67) eggs (mean±SD and range) during 2 and 4 days of contact with the host, respectively. Females that manifested egg retention, did not parasitize any host.

Three-way ANOVA showed that movement activity of T. principium females, estimated by the mean number of square borders crossed per one second of observation was strongly (F = 44.3, p<0.001) dependent on whether it parasitized before the test or not. Replicate (i.e. generation) influence was much weaker (F = 5.5, p<0.001), while the influence of the female’s age and the interaction of these factors were insignificant. By pooling data from of all replicates (Table 1), females retaining their eggs moved almost twice as much compared to ovipositing wasps.

Females that oviposited differ from those that retained eggs also in the rate of dispersal (i.e. increase in distance from the starting point). Both in 4 and 6 day old T. principium, the proportion of wasps that left small and large circles during one minute of observation in females that oviposited was much lower (Table 1). Mantel-Haenszel test showed high (p<0.01) significance of both differences.

An important point is that the differences in movement and dispersal activity between ovipositing and non-ovipositing females did not significantly increase with the number of eggs laid by ovipositing wasps. Pearson correlation coefficient between the number of square borders crossed per one second of observation and the number of eggs laid by ovipositing female before the test was insignificant (r = 0.07, n = 112). Mean number of eggs laid by ovipositing females that did not leave the small circle, females that left the small circle, and females that left the large circle was 34.3 ± 9.5 (16 – 55), 36.2 ± 12.0 (14 – 67), and 39.3 ± 12.5 (18 – 66), respectively. ANOVA showed that this slight increase was insignificant (p = 0.27).

## Discussion

The average fecundity of ovipositing wasps and the percentage of females that refused to parasitize are in agreement with our previous studies. At the beginning of oviposition, mean daily fecundity of T. principium females usually ranged up to 15 eggs and then sharply declined. The average longevity of T. principium females under experimental conditions was 6–8 days, although certain females survived over 12 days; the oviposition period lasted about 4 days. Although certain females started oviposition during first days after contact with the host, many of the wasps showed a delay in parasitization. That is why the total percentage of ovipositing females tended to increase with time (Reznik *et al.* 1998, 2001)

It is well known that movement activity of Trichogramma species (average speed, percentage of time spent moving, etc.) may depend on environmental conditions, primarily on temperature (Fournier and Boivin 2000; Suverkropp *et al.* 2001). Our data suggest that physiological state of female can also be important. Decrease in movement and dispersal in parasitizing females (as compared with those that refused to parasitize) is not just a direct consequence of time expenditure for handling and oviposition. In our experiments, in the absence of host eggs, females that had oviposited before the test show significant reduction of spontaneous walking activity and dispersal rate. It is necessary to stress that the observed effect cannot be considered as a direct arrestment reaction to the host (Gardner and Lenteren 1986; Nordlund 1994; Schmidt 1994) because no hosts were provided during the test period.

Slight, but significant difference in movement and dispersal activity were also recorded between replicates (generations). High inter-generational variability of Trichogramma was also noted by other authors (Schmuck *et al.*. 1996). We have studied cyclic dynamics of the percentage of ovipositing females and their mean fecundity in the generation sequence of two Trichogramma species. The results of this study suggest that these dynamics are at least partly determined by endogenous factors (see Reznik *et al.*. 1996 for more discussion). Similar endogenous changes in the percentage of diapausing pronymphs were earlier demonstrated for several Trichogramma species (Zaslavski and Umarova, 1990).

Although Trichogramma species fly in our experiments, flight was practically never observed supposedly because flight propensity is usually manifested at higher temperatures (Forsse *et al.* 1992; Prasad *et al.* 1999). Under natural conditions, long-range dispersal was often recorded, particularly in warm climate, e.g. T. ostriniae females were captured up to 180 m from the release point 6 days after release, and up to 230 m away in 21 days (Wright *et al.* 2001;Kuske *et al.* 2003). However, in many other field and laboratory studies the range of dispersal was several meters at most (Smith 1996; Brar  *>et al.* 2000; McGregor *et al.* 2000;Mehetre and Salunkhe 2000; Wang *et al.* 2000) suggesting that dispersal was mainly achieved by walking. Moreover, the same is true for host-oriented search (Noldus *et al.* 1991).

Reznik *et al.* (2001) demonstrated that oviposition by a group of simultaneously emerged females was uniformly distributed in time because of egg retention. Now, it is apparent that egg retention is accompanied with intensive movement activity, which increase the distance between progeny of a given female. Both temporal spread and dispersal in space can be considered as a strategy aimed at avoiding total elimination under unpredictable habitat changes (Danks 2002; Evans 2003). This mechanism seems to be even more adaptive when non-preferred (low quality) hosts are available, as in our experiments. Although S. cereallella is widely used for mass rearing of numerous Trichogramma species (Smith 1996), for some of these parasitoids this factitious laboratory host is near the lower threshold of acceptance. In our earlier studies with T. principium, egg retention was much less often observed when natural host (Noctuidae) eggs were provided. Work by other authors (e.g. Hofmann et al. 2001; Takada et al. 2001) with other Trichogramma species have also demonstrated that in spite of long term rearing on factitious hosts, natural hosts are still markedly preferred. Delayed parasitization of a poor quality host evidently results not only in temporal and spatial dispersal, but also in increasing the probability of finding other (better) hosts.

As noted earlier, delayed oviposition in the presence of non-preferred hosts was recorded in numerous insects (Donaldson and Walter 1988; Tepedino 1988; Kim 1999; Withers *et al.* 2000; Beck *et al.* 2001; Evans 2003). Thus, it is possible that the effect described in the present paper may be characteristic of other insect species.

## References

[i1536-2442-6-16-1-Beck1] Beck M. Reineke A. Lorenz H. Theopold U. Schmidt O. 2001 Two distinct reproductive strategies are correlated with an ovarian phenotype in co-existing parthenogenetic strains of a parasitic wasp Journal of Insect Physiology 47 1189 1195 12770197 10.1016/s0022-1910(01)00102-0

[i1536-2442-6-16-1-Bennett1] Bennett D. M. Reynolds K. T. Thomson L. J. Hoffmann A. A. 2002 Individual level trade-offs and artifacts in the egg parasitoid Trichogramma carverae (Hymenoptera, Trichogrammatidae) Annals of the Entomological Society of America 95 695 700

[i1536-2442-6-16-1-Brar1] Brar K. S. Khosa S. S. Sekhon B. S. 2000 Host searching capacity of laboratory reared and field collected populations of Trichogramma chilonis Ishii Journal of Biological Control 14 29 33

[i1536-2442-6-16-1-Carriere1] Carrière Y. Boivin G. 2001 Constraints on the evolution of thermal sensitivity of foraging in Trichogramma: genetic trade-offs and plasticity in maternal selection American Naturalist 157 570 581 10.1086/319931 18707263

[i1536-2442-6-16-1-Danks1] Danks H. V. 2002 The range of insect dormancy responses European Journal of Entomology 99 127 142

[i1536-2442-6-16-1-Dingle1] Dingle H. Winchell R. 1997 Juvenile hormone as a mediator of plasticity in insect life histories Archives of Insect Biochemistry and Physiology 35 359 373

[i1536-2442-6-16-1-Donaldson1] Donaldson J. S. Walter G. H. 1988 Effects of egg availability and egg maturity on the ovipositional activity of the parasitic wasp, Coccophagus atratus. Physiological Entomology 13 407 417

[i1536-2442-6-16-1-Evans1] Evans E. W. 2003 Searching and reproductive behaviour of female aphidophagous ladybirds (Coleoptera: Coccinellidae): a review European Journal of Entomology 100 1 10

[i1536-2442-6-16-1-Forsse1] Forsse E. Smith S. M. Bourchier R. S. 1992 Flight initiation in the egg parasitoid Trichogramma minutum: effects of ambient temperature, mates, food, and host eggs Entomologia Experimentalis et Applicata 62 147 154

[i1536-2442-6-16-1-Fournier1] Fournier F. Boivin G. 2000 Comparative dispersal of Trichogramma evanescens and Trichogramma pretiosum (Hymenoptera: Trichogrammatidae) in relation to environmental conditions Environmental Entomology 29 55 63

[i1536-2442-6-16-1-Gardner1] Gardner S. M. Lenteren J. C. 1986 Characterization of the arrestment responses of Trichogramma evanescens. Oecologia 68 265 270 10.1007/BF00384798 28310138

[i1536-2442-6-16-1-Hansen1] Hansen L. S. Jensen K. M. V. 2002 Effect of temperature on parasitism and host-feeding of Trichogramma turkestanika (Hymenoptera: Trichogrammatidae) on Ephesia kuehniella (Lepidoptera: Pyralidae) Journal of Economic Entomology 95 50 56 11942764 10.1603/0022-0493-95.1.50

[i1536-2442-6-16-1-Hoffmann1] Hoffmann M. P. Ode P. R. Walker D. L. Gardner J. van Nouhuys S. Shelton A. M. 2001 Performance of Trichogramma ostriniae (Hymenoptera: Trichogrammatidae) reared on factitious hosts, including the target host, Ostriniua nubilalis (Lepidoptera: Crambidae) Biological Control 21 1 10

[i1536-2442-6-16-1-Kim1] Kim J. K. 1999 Biological studies on Torymus sinensis Kamijo (Hymenoptera, Torymidae), a parasitoid of chestnut gall wasp Dryocosmus kuriphilus Yasumatsu (Hymenoptera, Cynipidae) Korean Journal of Applied Entomology 38 85 91

[i1536-2442-6-16-1-Kuske1] Kuske S. Widmer F. Edwards P. J. Turlings T. C. J. Babendreier D. Bigler F. 2003 Dispersal and persistence of mass released Trichogramma brassicae (Hymenoptera: Trichogrammatidae) in non-target habitats Biological Control 27 181 193

[i1536-2442-6-16-1-McGregor1] McGregor R. Caddick G. Henderson D. 2000 Egg loads and egg masses: parasitism of Choristoneura rosaceana eggs by Trichogramma minutum after inundative release in a commercial blueberry field BioControl 45 257 268

[i1536-2442-6-16-1-Mehetre1] Mehetre S. T. Salunkhe G. N. 2000 Studies on host searching capacity of Trichogramma pretiosum Riley, an egg parasitoid of tomato fruit borer Journal of Maharashtra Agricultural Universities 25 102 103

[i1536-2442-6-16-1-Monje1] Monje J. C. Zebitz C. P. W. Ohnesorge B. 1999 Host and host age preference of Trichogramma galloi and T. pretiosum (Hymenoptera: Trichogrammatidae) reared on different hosts Journal of Economic Entomology 92 97 103

[i1536-2442-6-16-1-Noldus1] Noldus L. P. J. J. van Lenteren J. C. Lewis W. J. 1991 How Trichogramma parasitoids use moth sex pheromones as kairomones: orientation behaviour in a wind tunnel Physiological Entomology 16 313 327

[i1536-2442-6-16-1-Nordlund1] Nordlund D. A. 1994 Habitat location by Trichogramma. In: Wajnberg E, Hassan SA, editors. *Biological control with egg parasitoids* 155 163 CAB International Wallingford, UK

[i1536-2442-6-16-1-Pavlik1] Pavlik J. 1993 Variability in the host acceptance of European corn borer, Ostrinia nubialis Hbn. (Lepidoptera, Pyralidae) in strains of the egg parasitoid Trichogramma spp. (Hymenoptera, Trichogrammatidae) Journal of Applied Entomology 115 77 84

[i1536-2442-6-16-1-Prasad1] Prasad R. P. Roitberg B. D. Henderson D. E. 1999 The effect of rearing temperature on flight initiation of Trichogramma sibiricum Sorokina at ambient temperatures Biological Control 16 291 298

[i1536-2442-6-16-1-Reznik1] Reznik S. Ya Umarova T. Ya 1991 Host population density influence on host acceptance in Trichogramma. Entomologia Experimentalis et Applicata 58 49 54

[i1536-2442-6-16-1-Reznik2] Reznik S. Ya Voinovich N. D. Umarova T. Ya 1996 Experimental studies of the dynamics of the percentage of ovipositing females and their fecundity in the generation sequence of Trichogramma (Hymenoptera, Trichogrammatidae) Entomological Review 76 138 143

[i1536-2442-6-16-1-Reznik3] Reznik S. Ya Umarova T. Ya Voinovich N. D. 1997 The influence of previous host age on current host acceptance in Trichogramma. Entomologia Experimentalis et Applicata 82 153 157

[i1536-2442-6-16-1-Reznik4] Reznik S. Ya Umarova T. Ya Voinovich N. D. 1998 Egg retention in the presence of a host in Trichogramma females Journal of Applied Entomology 122 555 559

[i1536-2442-6-16-1-Reznik5] Reznik S. Ya Voinovich N. D. Umarova T. Ya 2001 Long-term egg retention and parasitization in Trichogramma principium (Hymenoptera, Trichogrammatidae) Journal of Applied Entomology 125 169 175

[i1536-2442-6-16-1-Schmidt1] Schmidt J. M. 1994 Host recognition and acceptance by Trichogramma. In: Wajnberg E, Hassan SA, editors. *Biological control with egg parasitoids* 165 200 CAB International Wallingford, UK

[i1536-2442-6-16-1-Schmuck1] Schmuck R. Mager H. Künast Ch Bock K. D. Storck-Weyhermüller S. 1996 Variability in the reproductive performance of beneficial insects in standards laboratory toxicity assays - implications for hazard classification of pesticides Annals of Applied Biology 128 437 451

[i1536-2442-6-16-1-Silva1] Silva I. M. M. S. Stouthamer R. 1999 Do sympatric Trichogramma species parasitize the pest insect Helicoverpa armigera and the beneficial insect Chrysoperla carnea in different proportions Entomologia Experimentalis et Applicata 92 101 107

[i1536-2442-6-16-1-Smith1] Smith S. M. 1996 Biological control with Trichogramma: advances, successes, and potential of their use Annual Review of Entomology 41 375 406 10.1146/annurev.en.41.010196.002111 15012334

[i1536-2442-6-16-1-Suverkropp1] Suverkropp B. P. Bigler F. van Lenteren J. C. 2001 Temperature influences walking speed and walking activity of Trichogramma brassicae (Hymenoptera: Trichogrammatidae) Journal of Applied Entomology 125 303 307

[i1536-2442-6-16-1-Takada1] Takada Y. Kawamura S. Tanaka T. 2001 Host preference of Trichogramma dendrolimi (Hymenoptera: Trichogrammatidae) on its native host, Mamestra brassicae (Lepidoptera: Noctuidae) after 12 continuous generations on a factitious host Applied Entomology and Zoology 36 213 218

[i1536-2442-6-16-1-Tepedino1] Tepedino V. J. 1988 Aspects of host acceptance by Pteromalus venustus walker and Monodontomerus obsoletus Fabricius (Hymenoptera: Chalcidae), parasitoids of Megachile rotundata (Fabricius), the alfalfa leafcutting bee Pan-Pacific Entomologist 64 67 71

[i1536-2442-6-16-1-Wang1] Wang Z. Y. Zhou D. R. Hassan S. A. 2000 The dispersal distance and activity rhythm of Trichogramma ostriniae in greenhouse Acta Phytophylacica Sinica 27 17 22

[i1536-2442-6-16-1-Withers1] Withers T. M. Barton Browne L. Stanley J. 2000 How time-dependent processes can affect the outcome of assays In: Van Driesche R, Heard T, McClay A, Reardon R, editors. *Host specificity testing of exotic arthropod biological control agents: The biological basis for improvement in safety* 27 42 USDA Forest Health Technology Enterprise Team Morganton, West Virginia, USA

[i1536-2442-6-16-1-Wright1] Wright M. G. Hoffmann M. P. Chenus S. A. Gardner J. 2001 Dispersal behavior of Trichogramma ostriniae (Hymenoptera: Trichogrammatidae) in sweet corn fields: Implications for augmentative releases against Ostrinia nubilalis (Lepidoptera: Crambidae) Biological Control 22 29 37

[i1536-2442-6-16-1-Zaslavski1] Zaslavski V. A. Umarova T. Ya 1990 Environmental and endogenous control of diapause in Trichogramma species Entomophaga 35 23 29

